# Comparative analysis of onabotulinum toxin type-A injection techniques in older adults with blepharospasm: a retrospective cohort study

**DOI:** 10.3389/fneur.2025.1601911

**Published:** 2025-10-17

**Authors:** Turan Poyraz, Özgül Vupa Çilengiroğlu

**Affiliations:** ^1^Department of Elderly Care, Vocational Schools of Health Services, Izmir University of Economics, İzmir, Türkiye; ^2^Department of Statistics, Faculty of Science, Dokuz Eylül University, İzmir, Türkiye

**Keywords:** blepharospasm, botulinum neurotoxin, injection techniques, aging, Jankovic Scale

## Abstract

**Introduction:**

Benign essential blepharospasm (BEB) is a focal dystonia that can lead to functional blindness in older adults. While botulinum neurotoxin (BoNT) is widely utilized in the treatment of BEB, there remains a lack of consensus regarding the optimal injection technique. This study aims to compare the clinical outcomes and measurable scales associated with injections into the orbicularis oculi muscle at the pars pretarsalis (PPT) and pars preseptalis (PPS) regions, assessing their efficacy and side effects.

**Methods:**

The study included 32 patients diagnosed with BEB. Two injection techniques utilizing onabotulinum toxin type-A—PPT and PPS—were compared. The modified Jankovic Scale (mJS) was used to assess the clinical severity and frequency of blepharospasm. At the same time, the Blepharospasm Disability Scale evaluated the associated disabilities. The Schirmer I test was used to assess dry eye symptoms post-BoNT administration. Adverse effects were self-reported, and participants were examined at 1- and 3-month follow-up.

**Results:**

Both injection techniques resulted in significant improvements in the mJS severity and frequency scores, as well as the Blepharospasm Disability Scale (*p* < 0.05). Notably, improvements in ocular dryness were particularly pronounced at the 1-month mark (*p* < 0.05). The PPT injection technique displayed a superior safety profile, with the total number of adverse events statistically greater in the PPS group compared to the PPT group (*p* < 0.05).

**Conclusion:**

BoNT injections through both PPT and PPS techniques are effective in treating BEB in older adults. However, the PPT technique demonstrates a lower incidence of adverse events, rendering it a preferable choice for clinicians managing BEB.

## Introduction

Blepharospasm (BS) is the most common focal cranial dystonia in older adults. It is characterized by involuntary eyelid closure caused by spasmodic contractions of the periocular muscles. BS may start with increased eyelid blinking (1). BS most often occurs in the orbicularis oculi muscles (MOO) bilaterally, but it can rarely begin unilaterally (2). In the advanced stages of the disease, the eyelid may close permanently, leading to functional visual blindness that can seriously impact the patient’s work life, social interactions, and daily activities such as reading, writing, and driving (3).

BS symptoms can be triggered or worsened by stress, bright light, irritants to the eye (such as smoke or wind), anxiety, looking up or down, walking, reading, and fatigue. Conversely, symptoms may be alleviated by touching the forehead or eyelids, singing, talking, etc., which are called sensory tricks (geste antagonistique) (4).

BS is classified into primary and secondary BS. Secondary BS results from underlying conditions like multiple sclerosis, cerebral hemorrhage, or movement disorders. Besides known causes, infectious factors such as post-COVID-19 effects have also been reported (5). The cause and development of primary BS are largely unknown, which is why it is also called benign essential BS (BEB). The traditional view is that it results from hyperexcitability of brainstem interneurons caused by organic dysfunction of the basal ganglia (6). Studies have also shown that this phenomenon is related to the reduced activity of inhibitory neurons in the cerebral cortex caused by environmental factors and genetic predispositions (7–9). Increasing evidence has shown that the dysregulation of neurotransmitters such as dopamine, serotonin, and acetylcholine also plays an essential role in pathogenesis (9).

The prevalence of BEB varies by country, with about 16 to 133 cases per million. It was found to be more common in focal dystonias than in laryngeal and extremity dystonias, and less common than cervical dystonia (SD) according to North American and European studies (10, 11). It is more common among women and older adults (11).

Treatment options for BEB include medications and surgery, but these methods have limited effectiveness and variable success rates. They are also linked to many complications and side effects (12).

In 1985, Scott et al. (13) published the first reports on the use of onabotulinum toxin type-a (OnaBoNT-A) in the treatment of BEB. OnaBoNT-A was approved for BS in 1989 mainly based on the strong response observed in an open-label observational series (14). Since then, multiple reports on the efficacy and safety of BoNT-A treatment in BEB patients have been published. All these reports indicated that BoNT-A is effective for treating BEB with a low rate of adverse events (AEs), making BoNT-A the most commonly used treatment for BEB treatment (15, 16). Despite these studies, there is no consensus on the method of application of BoNT-A in the treatment of BEB.

Balance problems can occur in older adults with BEB. Postural stability (PS) declines in patients with BEB, especially during dual-task situations. A new study has shown that BoNT injections not only reduce eye contractions but also help improve patients’ balance issues (15, 16).

The MOO consists of three primary functional parts: the pars orbitalis (PO), pars preseptalis (PPS), and pars pretarsalis (PPT). The main motor functions of the PO and PPS mainly involve voluntary or spontaneous, sustained unilateral or bilateral narrowing or closing of the eyelids. The PPT section is primarily responsible for spontaneous, voluntary, or reflex blinking (17).

In our study, we aimed to compare the effects of changing the injection site on subjective and objective symptoms and AEs in older adults with BEB, where low doses and the same type of BoNT-A content were used.

## Methods

### Study design and patients

This was a retrospective, registry-based comparative cohort study using the Medifema Hospital Botulinum Toxin Clinic registry (January 2013–December 2023), which prospectively captures injections and outcomes. Patients were aged 65 or older with a diagnosis of isolated benign essential blepharospasm (BEB). Inclusion required three consecutive onabotulinumtoxinA (BoNT-A) sessions performed at the same center by the same neurologist using a single technique—pretarsal (PPT) or preseptal (PPS)—with no crossover, complete outcome data at baseline, Month 1, and Month 3, and the same BoNT-A formulation across sessions. Exclusion criteria included autoimmune diseases (e.g., Sjögren syndrome); initiation or change of central nervous system-active medication within the past 3 months (stable use beyond 3 months was permitted); mixed or secondary dystonia/hemifacial spasm; active ocular surface disease requiring treatment beyond artificial tears; and incomplete records. Allocation to PPT vs. PPS was nonrandom and determined by the treating neurologist based on eyelid anatomy and prior response.

### Data collection and outcomes

We reviewed all registry records with a diagnosis of benign essential blepharospasm (BEB). Records with hemifacial spasm (HFS), oromandibular dystonia, Meige syndrome, or cervical dystonia were excluded. Eligible cases had isolated BEB and received at least three consecutive BoNT-A sessions at the same center by the same neurologist, using a single technique—pretarsal (PPT) or preseptal (PPS)—without crossover. No patient met the clinical criteria for apraxia of eyelid opening or levator palpebrae inhibition. Outcomes were collected from the prospective registry, including the modified Jankovic Scale-Severity (mJS-S), modified Jankovic Scale-Frequency (mJS-F), and the Blepharospasm Disability Index (BSDI) at baseline, Month 1, and Month 3. Schirmer I testing was performed without topical anesthesia under standardized conditions (22 ± 2 °C, 40–60% humidity, low airflow); both eyes were measured over 5 min, and the average of both eyes was used for analysis. Demographics, disease duration, and AEs were also recorded; AEs were summarized as the presence of at least one event per patient during the observation period. All included patients received the same BoNT-A formulation (OnabotulinumtoxinA) across sessions.

### Ethical issues

The study was conducted in accordance with the Declaration of Helsinki and approved by the Bakırçay University Non-Interventional Research Ethics Committee (Approval No. 1250/1230; October 18, 2023). Written informed consent was obtained from all participants, and the committee authorized the use of de-identified data from the Medifema Hospital Botulinum Toxin Clinic registry.

### Survey tools

#### Evaluation of motor severity

##### Scales and scoring

Motor severity was assessed using the modified Jankovic Scale (mJS) and the Blepharospasm Disability Index (BSDI). The mJS includes two subscales analyzed separately: mJS-Severity (mJS-S), which rates symptom intensity, and mJS-Frequency (mJS-F), which rates occurrence. Each subscale is scored on five ordered categories (0–4), where 0 = no symptoms and 4 = most severe/most frequent; higher scores indicate worse severity or frequency.

##### BSDI administration

The BSDI measures disability across six daily activities (driving, reading, watching television, shopping, walking, and performing everyday tasks) with response options 0–4 (0 = no impairment; 4 = unable to perform due to disease), plus “not applicable” (N/A) when an activity does not apply to the patient. The BSDI score is calculated as the mean of applicable items (i.e., total score divided by the number of non-N/A items); the number of contributing items per patient is recorded. Higher BSDI scores indicate greater disability.

##### Timing and procedures

All scales were administered at baseline, Month 1, and Month 3 by the treating neurologist using standardized instructions and scoring manuals to ensure consistency.

#### Evaluation of lacrimal secretions/dry eyes

##### Procedures and timing

The Schirmer I test (without topical anesthesia) was performed at baseline, 1 month, and 3 months after onabotulinumtoxinA (BoNT-A) injection. Testing occurred in a quiet room under standardized ambient conditions (22 ± 2 °C; 40–60% relative humidity; low airflow; standard lighting) after roughly 10 min of seated acclimatization.

##### Technique

Commercially available 35-mm paper strips (tears topical) were placed at the lateral one-third of the lower eyelid margin on both sides. After 5 min, the length of wetting in millimeters was recorded for each eye. The main analytical measure was the average of both eyes; if data from one eye was missing, the value from the available eye was used based on a predetermined rule.

##### Interpretation

Dry eye was defined as less than 5 mm of wetting in 5 min; borderline was 5–10 mm. Higher values suggest increased tear production.

#### Botulinum neurotoxin injection protocol

All patients received bilateral onabotulinumtoxinA (BoNT-A) injections. Vials of 100 U (Botox^®^, Allergan/AbbVie) were reconstituted with 2 mL of 0.9% saline to achieve a final concentration of 5 U/0.1 mL. Injections were administered using a 30-gauge, 0.5-inch insulin syringe with patients in a supine position and eyelids gently closed.

##### Primary site map (used for both techniques)

We targeted five sites per eye: medial and lateral points on the upper eyelid, medial and lateral points on the lower eyelid, and one lateral orbital point. Each site received a single pass; no EMG guidance was used. The dose per site was 2.5 U, resulting in a bilateral total dose of 25 U when only primary sites were injected.

##### Technique-specific placement

- Pretarsal (PPT): injections were administered 2–3 mm from the lid margin along the pretarsal orbicularis muscle in the upper and lower lids, near the medial and lateral canthi.
- Preseptal (PPS): injections were placed 5–8 mm above or below the lid margin within the preseptal orbicularis of the upper and lower lids.

##### Additional predefined points and total dose

**Figure 1:**
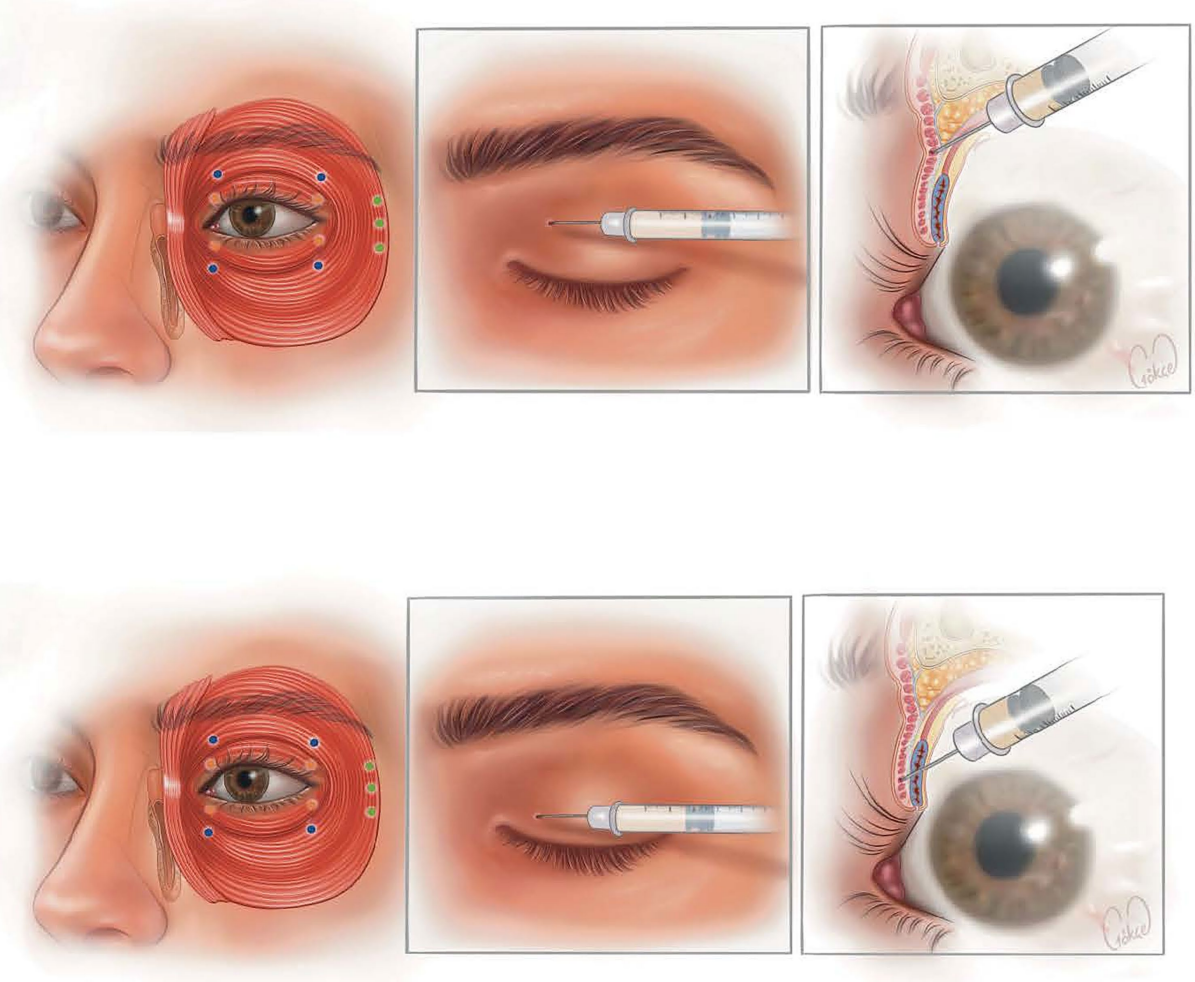
Injection techniques (This figure was created entirely by the authors). The first figure is PPS injection technique (the blue round dots indicate the injection points in PPS). The second figure is PPT injection technique (the orange round dots indicate the injection points in PPT). The green round dots indicate the injection points in the pars orbitalis (PO)’s lateral orbital portion.

In selected cases, predefined lateral periorbital and/or glabellar (corrugator/procerus) points were added based on spasm distribution, using 1.25–2.5 U aliquots per point. This explains the mean bilateral total doses per session observed for PPT: 38.6 ± 5.8 U and PPS: 36.9 ± 3.0 U in our cohort. The site map shown in the manuscript matches the schema above (Figure 1).

### Statistical analysis

Clinical variables were summarized as n (%), mean ± SD, or median (IQR), as appropriate. Baseline comparability between PPT and PPS was assessed using Welch’s *t*-test (or Mann–Whitney *U* test for non-normal data) and Fisher’s exact or *χ*^2^ tests for categorical variables; we also report standardized mean differences (SMDs) to quantify balance independent of sample size. The prespecified primary endpoint was the change in BSDI from baseline to Month 1. Secondary endpoints included mJS-S, mJS-F, Schirmer I, and the Month 3 timepoint for the same outcomes.

Longitudinal outcomes (mJS-S, mJS-F, BSDI, Schirmer) were analyzed using a two-way repeated-measures linear mixed-effects model with random intercepts for subjects: Outcome ~ Group (PPT vs. PPS) × Time (Baseline, Month 1, Month 3) + (1|Subject). We estimated Group × Time effects with Satterthwaite degrees of freedom and reported estimated marginal means (LS-means) with 95% confidence intervals, within-group changes (Δ), and between-group differences in change (ΔΔ) with 95% CIs. Because mixed models do not assume sphericity, Greenhouse–Geisser corrections and single-factor repeated-measures ANOVA were not used. Pairwise contrasts were limited to prespecified comparisons and adjusted for multiplicity (see below).

For the ordinal mJS subscales, we mainly treated scores as approximately interval (consistent with previous practice) and recognized this as a limitation; as a sensitivity analysis, we used cumulative-link mixed models. Responder analyses at Month 1 were predefined (≥1-point improvement for mJS-S; ≥0.5-point improvement for BSDI) and compared between groups with Fisher’s exact test; we report risk differences with Newcombe 95% CIs. AEs were summarized as patients with ≥1 AE (*n*, %) and compared using Fisher’s exact test; (event counts per patient were not recorded in the registry).

Distributional assumptions were checked using Shapiro–Wilk tests and Q–Q plots of model residuals; extremely small *p*-values are noted as *p* < 0.001. To address multiplicity across secondary endpoints and timepoints, we used the Holm step-down procedure and focused on effect sizes and precision rather than p-values alone. Analyses were carried out in SPSS v26 (MIXED, EMMeans); where applicable, results were confirmed with additional scripts.

### Sample size

This study investigates whether the injection technique (pars pretarsalis versus pars preseptalis) is associated with changes in disability and severity, while adjusting for potential confounders such as age, sex, disease duration, baseline BSDI, mJS-Severity/Function, baseline Schirmer I, total dose, and number of injection sites. The primary outcome measures the change in Blepharospasm Disability Index at 1 month (BSDI_M1). Secondary outcomes include changes in mJS-Severity/Function and Schirmer I at 1 and 3 months, as well as adverse event rates. Based on preliminary data from our retrospective cohort, the difference between techniques in BSDI_M1 showed a standardized mean difference of approximately *d* ≈ 0.88 (large). To be cautious, we planned for moderate to large effects (*d* = 0.60–0.80). *A priori* calculations for a two-arm comparison (two-sided *α* = 0.05) suggest that with *n* = 16 per group, the minimum detectable effect for 80% power is about *d* ≈ 0.99 without baseline adjustment; with ANCOVA/LMM baseline adjustment (assuming baseline → Month-1 correlation *ρ* ≈ 0.60), the 80% MDE decreases to approximately *d* ≈ 0.79. Under these assumptions, the power with *n* = 16 per group is approximately 62% for *d* = 0.80 without baseline adjustment and around 81% with baseline adjustment; for *d* = 0.88, the power is roughly 70% (unadjusted) and 88% (with baseline adjustment *ρ* ≈ 0.60). Therefore, the current sample size can detect large effects but may be underpowered for smaller ones. Missing data will be addressed under an MAR assumption using mixed-model likelihood, with multiple-imputation sensitivity analyses.

### Calculation formulas

We tested whether injection technique (pars pretarsalis vs. pars preseptalis) was associated with changes in disability and severity, adjusting for age, sex, disease duration, baseline BSDI and mJS-Severity/Function, baseline Schirmer I, total dose, and number of injection sites. The primary endpoint was change in Blepharospasm Disability Index at 1 month (ΔBSDI_M1). Secondary endpoints were changes in mJS-Severity/Function and Schirmer I at 1 and 3 months, and adverse-event rates. All tests were two-sided with *α* = 0.05.

Sample size and power calculations were anchored to the present dataset with equal allocation (*n* = 16 per group). Preliminary retrospective data indicated a between-technique effect on ΔBSDI_M1 corresponding to a standardized mean difference of approximately *d* ≈ 0.88. To remain conservative, we considered moderate-to-large effects (*d* = 0.60–0.80) and derived minimal detectable effects (MDEs) under a two-sample comparison of means. For equal group sizes, the required per-group sample size is given by Equation (1):


(1) $$n\_\mathrm{per}\;\text{group}=2\cdot {\left(Z\_\{1-\alpha /2\}+Z\_\{1-\beta \}\right)}^{²}/d^{²}$$

Equivalently, for fixed *n*, the detectable standardized effect is Equation (2):


(2) $$d\_\mathrm{MDE}=\surd \{\;2\cdot {\left(Z\_\{1-\alpha /2\}+Z\_\{1-\beta \}\right)}^{²}/n\_\mathrm{per}\;\text{group}\;\}\;$$

When baseline is included as a covariate in ANCOVA or linear mixed-effects models, variance is reduced by (1 − *ρ*^2^), where ρ is the baseline-follow-up correlation; hence, for fixed *n* the detectable effect scales as Equation (3):


(3) $$d\_\mathrm{MDE},\text{ANCOVA}=d\_\mathrm{MDE}\cdot \surd (1-\rho^{²})$$

Using *Z*_{1 − 0.05/2} = 1.96 and *Z*_{1 − 0.20} = 0.84 (80% power), with *n* = 16 per group the unadjusted MDE is *d* ≈ 0.99. Assuming *ρ* ≈ 0.60 for baseline → Month-1 BSDI, the ANCOVA-adjusted MDE is *d* ≈ 0.79. Under these assumptions, the achieved power with *n* = 16 per group is ≈62% for *d* = 0.80 without baseline adjustment and ≈81% with baseline adjustment; for *d* = 0.88 the achieved power is ≈70% (unadjusted) and ≈88% (with baseline adjustment). Missing data were addressed under a missing-at-random (MAR) assumption using mixed-model likelihood, with multiple-imputation sensitivity analyses.

## Results

### Cohort and flow

**Figure 2:**
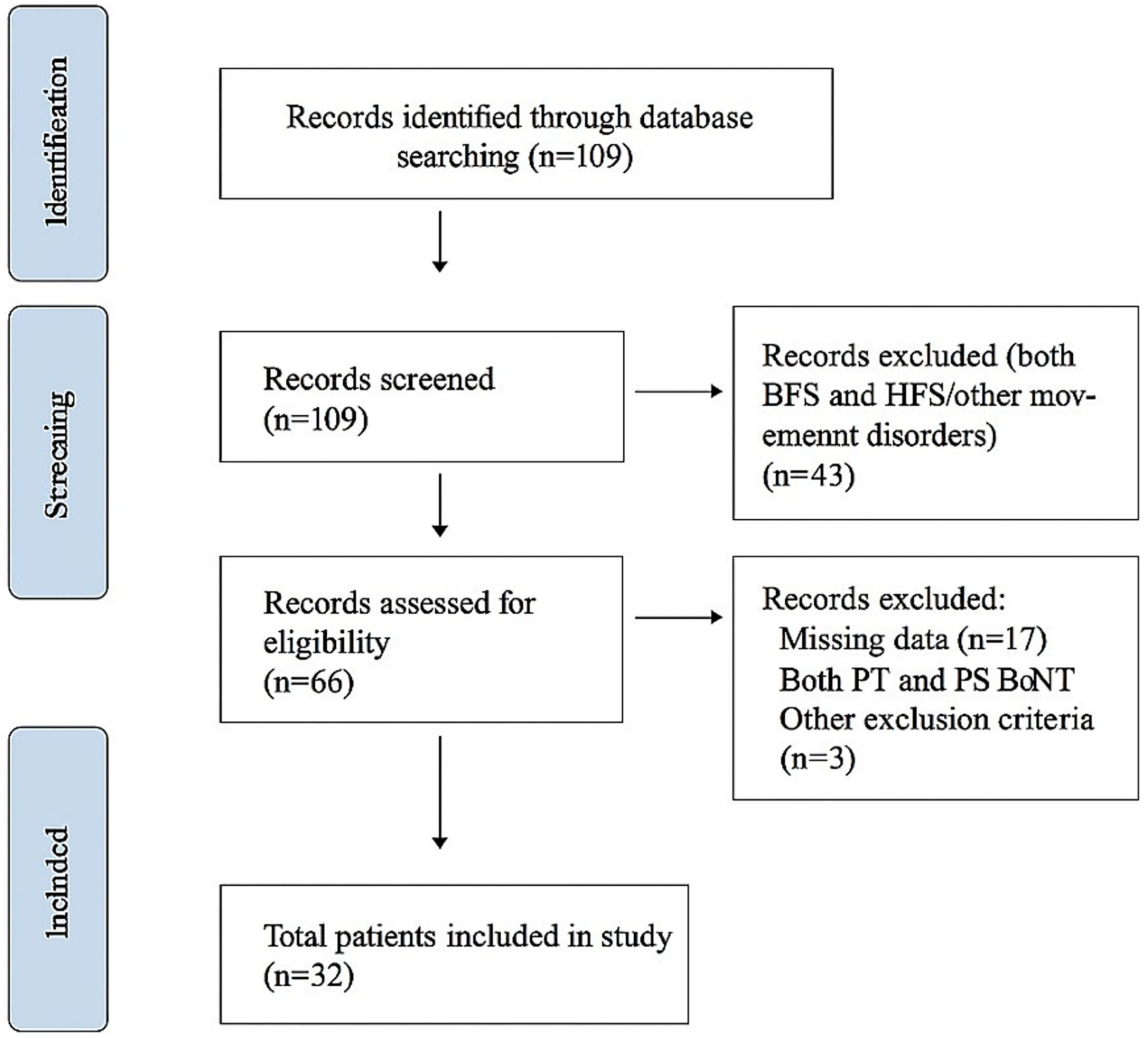
The flowchart of the study.

This study aimed to assess how the application method of BoNT-A treatment affects involuntary eye contractions, specifically examining test parameters related to the mJS-S and mJS-F of the disease, as well as clinical parameters like BSDI and the Schirmer scale. A total of 32 patients were evaluated at the Medifema Hospital BoNT Clinic in Turkey from January 2013 to December 2023. During the period, 32 patients who met the inclusion criteria were included in the study. Screening, eligibility, inclusion, and reasons for exclusion are summarized in the flow diagram (Figure 2).

### Baseline comparability

Normality was assessed with the Shapiro–Wilk test (and Q–Q inspection); only age approximated a normal distribution (*p* > 0.05). Accordingly, we used Welch’s *t*-test for age and Mann–Whitney *U* for other continuous variables. No between-group differences were detected between PPT and PPS for continuous characteristics (all *p* > 0.05), including age, time from complaint to diagnosis (months), time from diagnosis to BoNT-A (days), baseline mJS-S/mJS-F, BSDI, Schirmer I, and bilateral total BoNT-A dose per session. Detailed results are presented in Table 1 as mean ± SD [or median (IQR) when non-normal], with *p*-values and standardized mean differences (SMDs).

**Table 1:** Baseline continuous characteristics by technique (PPT vs. PPS).

Variable	PPT (mean ± SD)	PPS (mean ± SD)	*p*-value^a^	SMD	*N* (PPT/PPS)
Age (years)	68.38 ± 2.36	68.50 ± 3.01	0.897	0.05	16/16
Time from complaint to diagnosis (months)	21.12 ± 18.28	16.56 ± 9.99	0.390	0.31	16/16
Time from diagnosis to BoNT-A (days)	27.38 ± 28.84	29.56 ± 33.43	0.844	0.07	16/16
Number of injections (lifetime)	15.44 ± 2.31	14.75 ± 1.18	0.300	0.37	16/16
Total dose per session (U)^b^	38.59 ± 5.77	36.88 ± 2.96	0.300	0.37	16/16
mJS-S (baseline)	2.56 ± 0.96	2.50 ± 0.63	0.830	0.08	16/16
mJS-F (baseline)	2.75 ± 0.68	2.81 ± 0.54	0.777	0.10	16/16
BSDI (baseline)	2.62 ± 0.92	2.54 ± 0.64	0.782	0.10	16/16
Schirmer I (mm, baseline)	8.59 ± 4.41	7.50 ± 3.04	0.422	0.29	16/16

Values are mean ± SD.

PPT, pretarsal technique; PPS, preseptal technique; BoNT-A, botulinum toxin type A; U, onabotulinumtoxinA units; mJS-S, modified Jankovic Scale-Severity; mJS-F, Modified Jankovic Scale-Frequency; BSDI, Blepharospasm Disability Index; SD, standard deviation; SMD, standardized mean difference.

a Welch’s *t*-test. SMD = standardized mean difference.

b Dose reported as U (onabotulinumtoxinA units).

### Categorical characteristics

Group comparisons for categorical variables [e.g., sex, initial form (unilateral/bilateral), sensory trick, triggers: stress/bright light, dry eye symptom, pain] were performed using *χ*^2^ or Fisher’s exact tests as appropriate. No associations were observed between technique (PPT vs. PPS) and these categorical characteristics (all *p* > 0.05). Counts and proportions with corresponding *p*-values and SMDs (proportions) are summarized in Table 2.

**Table 2:** Baseline categorical characteristics by technique (PPT vs. PPS).

Variable	PPT *n*/*N* (%)	PPS *n*/*N* (%)	Test	*p*-value	SMD (prop.)	*N* (PPT/PPS)
Sex (female)	—	—	Fisher	1.000	—	0/0
Marital status (yes)	12/16 (75.0%)	12/16 (75.0%)	Fisher	1.000	0.00	16/16
Initial form (bilateral)	7/16 (43.8%)	7/16 (43.8%)	Chi-square	1.000	0.00	16/16
Sensory trick (yes)	13/16 (81.2%)	10/16 (62.5%)	Fisher	0.433	0.42	16/16
Increases with stress (yes)	14/16 (87.5%)	16/16 (100.0%)	Fisher	0.484	0.52	16/16
Increases with bright light (yes)	10/16 (62.5%)	12/16 (75.0%)	Fisher	0.704	0.27	16/16
Excessive blinking (yes)	16/16 (100.0%)	16/16 (100.0%)	Fisher	1.000	—	16/16
Eyelid spasm (yes)	12/16 (75.0%)	10/16 (62.5%)	Fisher	0.704	0.27	16/16
Dry eye symptom (yes)	11/16 (68.8%)	12/16 (75.0%)	Fisher	1.000	0.14	16/16
Pain (yes)	6/16 (37.5%)	3/16 (18.8%)	Fisher	0.433	0.42	16/16

Values are *n*/*N* (%). Test = Fisher’s exact or chi-square as appropriate. SMD (prop.) = standardized mean difference for proportions.

PPT, pretarsal technique; PPS, preseptal technique; *n*, number with the characteristic; *N*, number with non-missing data; *χ*^2^, chi-square; SMD, standardized mean difference.

### Primary endpoint—BSDI change at Month 1

Both techniques showed significant improvement in the Blepharospasm Disability Index (BSDI) from baseline to Month 1, with some decline toward baseline by Month 3 (Supplementary Tables S1–S4). The main analysis used a linear mixed-effects model (Outcome ~ Group × Time + (1|Subject)). The Group × Time interaction for BSDI was not significant (e.g., *p* = 0.429), and the difference in change between groups (ΔΔ, PPT − PPS) was 0.26 (95% CI − 0.23 to 0.76) at Month 1 and 0.09 (95% CI − 0.22 to 0.40) at Month 3. This indicates no statistically or clinically meaningful advantage of one technique over the other in reducing disability. An exploratory, unadjusted two-sample comparison showed a difference at Month 1 (original *t*-test *p* = 0.016), but this did not hold up in the prespecified mixed-effects model or after accounting for multiple comparisons.

### mJS subscales—severity and frequency

Modified Jankovic Scale-Severity (mJS-S) and -Frequency (mJS-F) scores decreased significantly at Month 1 and partially rebounded by Month 3 in both groups, consistent with the expected timeline of onabotulinumtoxinA. In mixed-effects models, the Group × Time interaction for mJS-S was not significant (e.g., *p* = 0.179), and no consistent differences emerged between groups for mJS-F (Supplementary Tables S1–S4). Responder analyses predefined a ≥ 1-point improvement in mJS-S and a ≥ 0.5-point improvement in BSDI at Month 1; responder rates were complete in both groups (PPT 16/16 vs. PPS 16/16; Fisher *p* = 1.000).

### Schirmer I (tear production)

Schirmer I values (no anesthesia) increased at Month 1 compared to baseline in both groups and moved closer to baseline by Month 3. Group differences were small and not clinically significant in mixed-effects estimates (Supplementary Tables S1–S4). Testing conditions and analytical methods (bilateral measurement; mean of both eyes; predefined single-eye rule) are detailed in Methods.

### Ancillary and sensitivity analyses

Estimated marginal means (LS-means) with 95% CIs for Group × Time cells are provided in Supplementary Tables S1–S4. Sensitivity analyses excluding dose outliers (1.5 × IQR) yielded similar results in direction [e.g., exploratory ΔΔBSDI at Month 1 1.21 (95% CI 0.27, 2.15)], reflecting the influence of small-sample variability; these are labeled exploratory and do not change the overall conclusions.

### Adverse events

AEs were rare. The registry recorded whether each patient experienced at least one AE rather than counting every individual event; therefore, we report the number and percentage of patients with at least one AE (*n*, %) and clearly specify the denominators (Table 3): PPT 4/16 (25.0%) and PPS 2/16 (12.5%). Due to the small sample sizes, differences between groups should be interpreted with caution.

**Table 3:** Adverse events (patients with ≥1 AE) and type-specific events by technique.

Adverse event	PPT *n*/*N* (%)	PPS *n*/*N* (%)	Test	*p*-value	*N* (PPT/PPS)
Any AE (≥1)	4/16 (25.0%)	2/16 (12.5%)	Fisher	0.654	16/16
Ptosis	1/16 (6.2%)	1/16 (6.2%)	Fisher	1.000	16/16
Diplopia	0/16 (0.0%)	0/16 (0.0%)	Fisher	1.000	16/16
Lagophthalmos	2/16 (12.5%)	2/16 (12.5%)	Fisher	1.000	16/16

AEs are captured as presence/absence per patient in the registry; per-patient event counts and severity grades were not available. Fisher’s exact test was used due to small cell counts.

AE, adverse event; PPT, pretarsal technique; PPS, preseptal technique; *n*, number with the event; *N*, number with non-missing data.

### mJS subscales (severity and frequency)

**Figure 3:**
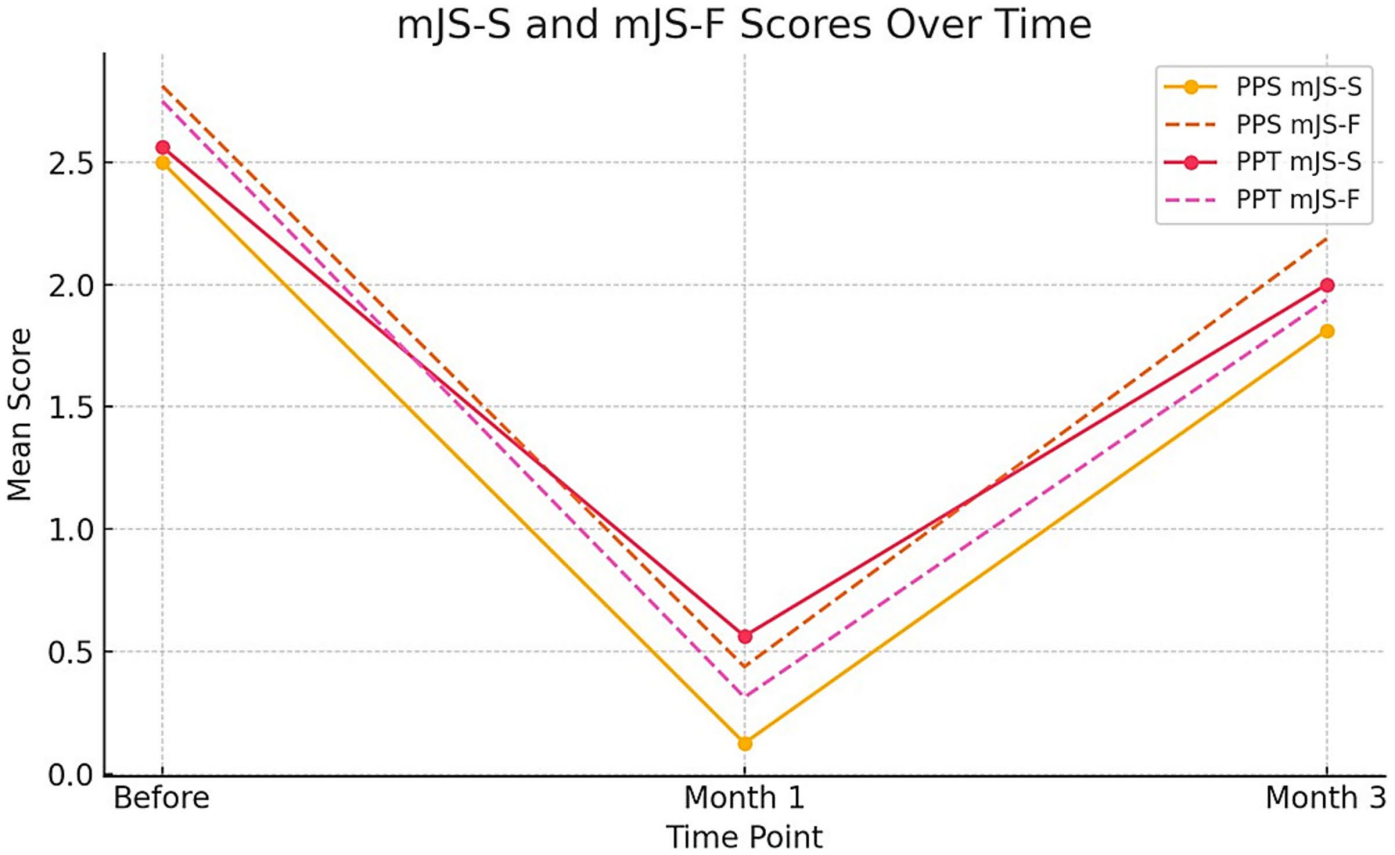
Line graph illustrating the mean mJS-S (modified Jankovic Scale-Severity) and mJS-F (modified Jankovic Scale-Frequency) scores for PPS and PPT groups over three time points: before treatment, 1 month after treatment, and 3 months after treatment. Solid lines represent mJS-S scores and dashed lines represent mJS-F scores.

The modified Jankovic Scale-Severity (mJS-S) and -Frequency (mJS-F) were evaluated at Baseline, Month 1, and Month 3 using ordinal scales of 0–4. As expected for onabotulinumtoxinA, both groups experienced a significant decrease at Month 1, with partial recovery by Month 3. The primary longitudinal analysis, pre-specified, employed a linear mixed-effects model (Outcome ~ Group [PPT vs. PPS] × Time + (1|Subject)). In this model, the Group × Time interaction was not significant for either mJS-S or mJS-F, indicating no difference in progression between techniques over time. Consistent with the mixed-effects results, we show a single summary figure (Figure 3) displaying LS-means (least-squares means; model-based estimated marginal means) with 95% CIs for mJS-S, mJS-F, and BSDI across Baseline, Month 1, and Month 3 by technique (PPT vs. PPS). For transparency, category distributions (0–4) for mJS-S and mJS-F at each time point and by technique are summarized in Supplementary Tables S5, S6, while model-based LS-means with 95% CIs are reported in Supplementary Tables S1–S4. An exploratory comparison of Month-1 mJS-S category distributions revealed a trend toward lower scores in the PPT group (*p* = 0.067 by chi-square), but this did not reach the two-sided *α* = 0.05 threshold and was not considered evidence of a between-group difference after adjusting for multiplicity and the ordinal scale. Therefore, inference depends on the mixed-effects estimates.

### LS-means clarification

We report LS-means (least-squares means; i.e., model-based estimated marginal means) with 95% confidence intervals for each Group × Time cell (Supplementary Tables S1–S4). LS-means and their 95% CIs overlapped across PPT and PPS at each time point, consistent with the non-significant Group × Time tests.

The statistical differences between the averages of the mJS-S and mJS-F test measurements taken at different time points (before, 1st month, 3rd month) for PPS application were determined using repeated measures ANOVA. Similarly, the difference between the averages of the mJS-S and mJS-F test measurements at different time points for PPT applications was statistically significant (all *p*-values <0.05). The highest mJS-S test measurement was observed before the test in the PPS and PPT applications, while the lowest was recorded in the first month. Likewise, the highest mJS-F test measurement was observed before the test in the PPS and PPT applications, and the lowest was recorded in the first month after the test (Table 4).

**Table 4:** mJS-S and mJS-F by technique and time (descriptive means ± SD; within-group changes).

Outcome	Group	Baseline mean ± SD	Month 1 mean ± SD	Month 3 mean ± SD	Δ Month 1 − Baseline	Δ Month 3 − Baseline	*N*
mJS-S	PPT	2.56 ± 0.96	0.56 ± 0.63	2.00 ± 0.73	−2.00 ± 0.73	−0.56 ± 0.51	16
mJS-S	PPS	2.50 ± 0.63	0.12 ± 0.34	1.81 ± 0.66	−2.38 ± 0.62	−0.69 ± 0.48	16
mJS-F	PPT	2.75 ± 0.68	0.31 ± 0.48	1.94 ± 0.68	−2.44 ± 0.63	−0.81 ± 0.40	16
mJS-F	PPS	2.81 ± 0.54	0.44 ± 0.51	2.19 ± 0.54	−2.38 ± 0.50	−0.62 ± 0.50	16

Values are descriptive means. Within-group changes (Δ) are Month 1/3 minus Baseline. Primary inference is based on mixed-effects models; LS-means with 95% CIs are provided in Supplementary Tables S2, S3; Group × Time interactions were not significant.

mJS-S, Modified Jankovic Scale-Severity (0–4); mJS-F, Modified Jankovic Scale-Frequency (0–4); PPT, pretarsal technique; PPS, preseptal technique; SD, standard deviation; Δ, within-group change; LS-means, least-squares means (estimated marginal means); CI, confidence interval.

Understanding the difference between PPS and PPT applications over time is crucial. Therefore, in the independent two-sample *t*-test conducted to compare PPS and PPT applications across the pretest, 1st month posttest, and 3rd month posttest values, a statistically significant difference was observed only in the 1st month of the BSDI test (*p* = 0.016 < 0.05). A graphical summary of the changes between the means of the mJS-S and mJS-F test measurements is shown in Supplementary Figure S1.

### BSDI and Schirmer over time

Differences in BSDI and Schirmer I across Baseline, Month 1, and Month 3 were analyzed using a two-way linear mixed-effects model (Outcome ~ Group [PPT vs. PPS] × Time + (1|Subject)) (Table 5). Mixed models do not assume sphericity; therefore, Greenhouse–Geisser corrections were not applied. A strong main effect of Time was observed for both endpoints (all *p* < 0.001). Consistent with the original descriptive values, BSDI was highest at baseline (PPS 2.61; PPT 2.54) and lowest at Month 1 (PPS 0.47; PPT 0.13), with partial recovery by Month 3. For Schirmer I, values increased at Month 1 compared to baseline (PPS 13.00 mm; PPT 11.81 mm vs. baseline PPS 8.59 mm; PPT 7.50 mm). The Group × Time interaction was not significant for either BSDI or Schirmer, indicating no different trajectories between PPS and PPT. *Post-hoc* Tukey tests were not used in the mixed-model framework; instead, we report least-squares means (LS-means; estimated marginal means) with 95% CIs and prespecified contrasts (Supplementary Tables S1–S4). All *p*-values are two-sided and are reported as *p* < 0.001 where applicable.

**Table 5:** BSDI and Schirmer I—Group × Time means, within-group changes (Δ), and between-group differences (ΔΔ).

Outcome	Statistic	Group	Baseline mean ± SD (95% CI)	Month 1 mean ± SD (95% CI)	Month 3 mean ± SD (95% CI)	Δ Month 1 − Baseline (mean ± SD; 95% CI)	Δ Month 3 − Baseline (mean ± SD; 95% CI)	*N*
BSDI	Means & Δ	PPT	2.62 ± 0.92 (2.13, 3.10)	0.47 ± 0.49 (0.21, 0.73)	2.11 ± 0.71 (1.73, 2.49)	−2.15 ± 0.75 (−2.55, −1.75)	−0.51 ± 0.43 (−0.74, −0.28)	16
BSDI	Means & Δ	PPS	2.54 ± 0.64 (2.20, 2.88)	0.13 ± 0.22 (0.01, 0.25)	1.94 ± 0.63 (1.60, 2.27)	−2.41 ± 0.60 (−2.73, −2.09)	−0.60 ± 0.43 (−0.83, −0.37)	16
BSDI	ΔΔ (PPT − PPS)	—	—	—	—	Month 1: 0.26 (−0.23, 0.76)	Month 3: 0.09 (−0.22, 0.40)	—
Schirmer I (mm)	Means & Δ	PPT	8.59 ± 4.41 (6.24, 10.95)	13.00 ± 4.03 (10.85, 15.15)	11.09 ± 3.87 (9.03, 13.15)	4.41 ± 2.03 (3.32, 5.49)	2.50 ± 1.88 (1.50, 3.50)	16
Schirmer I (mm)	Means & Δ	PPS	7.50 ± 3.04 (5.88, 9.12)	11.81 ± 2.97 (10.23, 13.39)	9.56 ± 2.91 (8.01, 11.11)	4.31 ± 1.97 (3.26, 5.36)	2.06 ± 1.55 (1.24, 2.89)	16
Schirmer I (mm)	ΔΔ (PPT − PPS)	—	—	—	—	Month 1: 0.09 (−1.35, 1.54)	Month 3: 0.44 (−0.81, 1.68)	—

Means are descriptive summaries. Δ = within-group change from baseline. ΔΔ = between-group difference in change with Welch 95% CI. Primary inference is based on mixed-effects models (Results); Group × Time interactions were not significant for BSDI or Schirmer I.

BSDI, Blepharospasm Disability Index; Schirmer I, Schirmer tear test without anesthesia (mm/5 min); PPT, pretarsal technique; PPS, preseptal technique; Δ, within-group change; ΔΔ, between-group difference in change; CI, confidence interval; SD, standard deviation.

The difference between PPS and PPT applications was only significant during the first month of the BSDI test (*p* = 0.016 < 0.05) (Supplementary Figure S2).

While an exploratory, unadjusted two-sample comparison suggested a difference at Month 1 for the BSDI (*p* = 0.016), our prespecified primary analysis, using a linear mixed-effects model (which accounts for within-subject correlation and evaluates the Group × Time interaction), found no significant interaction for the BSDI (*p* = 0.429). The between-group difference in change at Month 1 was slight and imprecisely estimated (ΔΔBSDI = 0.26, 95% CI − 0.23 to 0.76) and did not survive multiplicity control; the Month-3 estimate was similarly non-significant (ΔΔBSDI = 0.09, 95% CI − 0.22 to 0.40). LS-means (least-squares means) with 95% CIs overlapped across techniques at each time point. Accordingly, we refrain from inferring the superiority of either method: both PPT and PPS were associated with notable improvement at Month 1 and partial return by Month 3. Given the modest sample size and nonrandom allocation, these findings should be interpreted as associations in comparative effectiveness; confirmatory randomized trials are warranted.

## Discussion

BEB is regarded as one of the most problematic movement disorders in older adults because it affects daily life and causes cosmetic concerns. Since the adoption of BoNT-A for the treatment of BEB in 1985, extensive open-label studies have demonstrated its efficacy and safety. To date, two A subtypes (onabotulinum toxin and incobotulinum toxin) have been approved for clinical use in the treatment of BEB (18).

The vast majority of patients included in the study were women, a finding that aligns with previous epidemiological studies. The initial presentation of BEB usually occurs bilaterally in the fifth decade of life, although the onset is unilateral in most patients in this study. This may be due to the neglect of mild symptoms in the eye with no complaints.

Sensory trickery was observed in most patients. The most common complaints before BoNT treatment included excessive blinking, stress-triggered issues, dry eyes, sensitivity to bright light, eyelid contractions, and pain. Overall, fewer AEs occurred in the PPT group. The findings of the present study were consistent with those of previous studies (19–21).

The mJS-S and mJS-F scales measure BEB severity and symptom frequency. In our study, both the PPT and PPS methods showed significant improvements in scale scores at 1 month. Improvement continued at 3 months but was not statistically significant. Both methods showed a notable decrease in the BSDI scale at 1 month, with this considerable reduction lasting at 3 months, although less pronounced in the PPT group. Previous studies highlighted better outcomes with the PPT technique regarding severity, frequency, and disability scales (19, 22). Our study revealed that these scales were similarly effective in both groups.

Dry eye symptoms have been reported in many patients with BEB (23–25). Lacrimal drainage capacity is affected by the blink rate. The injection of BoNT, especially when applied to the medial lower eyelid, prevents contraction of the orbicularis oculi muscle, causing a decrease in the effect of the lacrimal pump. Thus, lubrication of the ocular surface is improved (25, 26).

In this study, a clinical evaluation of the change in dry eye symptoms was performed with the Schirmer test. In both PPS and PPT applications, the measurements, which were shorter before the procedure, were statistically significantly prolonged, especially in the 1st month of BoNT application. The prolongation continued into the third month, although it showed a lower measurement value than in the first month. The clinical data from the Schirmer test were evaluated in accordance with previous studies (27). As a result of self-reports parallel to this clinical test, a statistically significant improvement in dry eye volume was observed in both the PPT and PPS groups.

BEB mainly affects the orbicularis oculi muscle. These muscles are divided into two main groups: the orbital and palpebral parts. The palpebral part is further divided into two sections: the pars preseptalis and the pars pretarsalis. Although both sections are responsible for eyelid closure, there are histological differences. The pretarsal section contains more skeletal muscle and has a higher innervation density per region than the preseptal section (28). Theoretically, this would be expected to be more responsive to BoNT treatment in PPT (19, 29). Additionally, the PPT contains a higher proportion of type 2 muscle fibers, which are shorter in length, thereby allowing greater BoNT penetration. In contrast, the PPS subpart contains significantly larger type 1 fibers (28, 30, 31).

In our study, we examined the overall occurrence of AEs and self-reported outcomes such as ptosis, epiphora, ecchymosis, irritation, diplopia/blurred vision, and lagophthalmos. Similar to previous research, PPS injection was associated with a higher risk of AEs, including ptosis, irritation, ecchymosis, and epiphora (20, 32). In both application methods, no diplopia or blurred vision was observed, unlike the studies conducted by Jankovic et al. (19) and Albanese et al. (33). This may be related to the dose we used. In addition, it may also be related to the application to two different pretarsal areas, more medial and lateral, unlike the single injection point in the lower eyelid pretarsal segment of Çakmur et al. (20) Again, the toxin used in the study by Aramideh et al. (34) was abobotulinumtoxin-A, which may be related to the spread of the toxin due to changes in diffusion rate caused by the molecular differences of the toxin. The PPS is located closer to the levator palpebrae muscle, which assists with eyelid elevation, and the injection of BoNT at this location may cause ptosis. Histologically, the PPS contains more fatty tissue, resulting in inadequate support for the eyelid muscles (29, 35). Previous studies have reported ecchymosis with the PPS injection technique. This observation may be explained by the abundance of underlying subdermal capillaries in the PPS. One of our patients had an ecchymosis associated with the procedure. In our study, lagophthalmos was observed at the same rate (6.25%) with both injection methods. Different results have been reported regarding the relationship between the injection method and lagophthalmos. In a randomized controlled study conducted by Teekaput et al. (22) in 2021, it was reported that measured lagophthalmos was found more in the PPT injection method. A prospective study using electromyographic methods to compare the effectiveness of injection sites revealed that a smaller amount of BoNT-A injected into the PPT was more effective than a larger amount injected into the PPS (36). In a single-blind, randomized controlled study by Jankovic in patients with BEB or apraxia of eyelid opening, BoNT-A injection into the right eye PPT and the left eye PPS showed that both injection sites were similarly effective (37). Both studies suggested that lagophthalmos could be reduced if lower doses of neurotoxin were applied to the PPT or if injections were made slightly further from the eyelid margin. In our study, the lack of a difference between the application methods and the relatively low rate of lagophthalmos may be related to the fact that the injection sites were kept further from the lateral and medial eyelid margins, both in the upper and lower eyelids. Our study results demonstrated that all problems associated with BoNT were related to the injection method.

In summary, the PPT approach was associated with a lower incidence of AEs, consistent with previous studies (19–22, 29, 32, 38–40). In addition, studies analysing differences between BoNT types have reported no significant difference in the occurrence of side effects (21, 29, 41, 42). We did not evaluate this because we used the same type of BoNT in our study design.

This study has several limitations. First, it is a single-center, retrospective, registry-based comparative cohort with small groups (*n* = 16 per arm) and nonrandom allocation to PPT versus PPS, which introduces potential confounding by indication and limits causal inference and generalizability. Second, outcomes were evaluated by the treating neurologist (unblinded); although standardized scripts were used, observer bias cannot be ruled out. Third, the registry recorded AEs as presence or absence per patient (without detailed counts or severity grading), and the study lacked sufficient power for safety endpoints and subgroup analyses. Fourth, the ordinal mJS-S/mJS-F scales were analyzed as approximately interval in the primary models; while this is consistent with prior practice, it remains an assumption (addressed in sensitivity analyses). Fifth, inclusion required complete data at baseline, Month 1, and Month 3, which may introduce selection bias. Finally, we used a single BoNT-A formulation (onabotulinumtoxinA) across sessions; this ensures internal consistency but prevents product-to-product comparisons warranted.

## Conclusion

In this retrospective, registry-based comparison of older adults with isolated BEB, both pretarsal (PPT) and preseptal (PPS) onabotulinumtoxinA injections showed significant improvement at Month 1, with partial decline by Month 3. Prespecified mixed-effects models indicated no significant Group × Time differences for BSDI, mJS-S, or mJS-F, suggesting similar effectiveness over time. Adverse events were rare; although differences between groups were small and imprecise, clinicians can reduce lagophthalmos by using the lowest effective dose and administering PPT injections about 2–3 mm from the lid margin. Due to nonrandom allocation and limited safety data, well-powered randomized trials are needed to determine whether meaningful safety or durability differences exist between the treatments techniques.

## Data Availability

The raw data supporting the conclusions of this article will be made available by the authors, without undue reservation.

## Supplementary material

SUPPLEMENTARY FIGURE S1 The statistical differences between the means of the mJS test measurements taken at different time periods (before, 1st month, 3rd month) for the PPS and PPT applications. **(A)** The statistical differences between the means of the mJS-S test measurements taken at different time periods (before, 1st month, 3rd month) for PPS and PPT application. **(B)** The statistical differences between the means of the mJS-F test measurements taken at different time periods (before, 1st month, 3rd month) for PPS and PPT application. mJS-S, modified Jankovic Scale Severity; mJS-F, modified Jankovic Scale Frequency; PS, preseptalis; PT, pretarsalis; M, Month.

SUPPLEMENTARY FIGURE S2 The statistical differences between the means of the BSDI and Schirmer test measurements taken at different time periods (before, 1st month, 3rd month) for PPS and PPT application. **(A)** The statistical differences between the means of the BSDI measurements taken at different time periods (before, 1st month, 3rd month) for PPS and PPT application. **(B)** The statistical differences between the means of the Schirmer test measurements taken at different time periods (before, 1st month, 3rd month) for PPS and PPT application. BSDI, Blepharospasm Disability Index; PS, preseptalis; PT, pretarsalis; M, Month; mm, millimeters; min, minutes.
