# Supplementary material for: Comparative analysis of onabotulinum toxin type-A injection techniques in older adults with blepharospasm: a retrospective cohort study
Source: Front Neurol. 2025 Oct 17;16:1601911. doi: 10.3389/fneur.2025.1601911 (PMC12576801; doi:10.3389/fneur.2025.1601911)
Supplement: Supplementary file 1 [file Table_1.docx]

**Table S1. BSDI — Estimated means and changes by Group × Time**

| Group | Baseline mean ± SD (95% CI) | Month 1 mean ± SD (95% CI) | Month 3 mean ± SD (95% CI) | N |
| --- | --- | --- | --- | --- |
| PPT | 2.62 ± 0.92 (2.13, 3.10) | 0.47 ± 0.49 (0.21, 0.73) | 2.11 ± 0.71 (1.73, 2.49) | 16 |
| PPS | 2.54 ± 0.64 (2.20, 2.88) | 0.13 ± 0.22 (0.01, 0.25) | 1.94 ± 0.63 (1.60, 2.27) | 16 |

| Timepoint | Δ (PPT) mean ± SD (95% CI) | Δ (PPS) mean ± SD (95% CI) | ΔΔ (PPT − PPS) (95% CI) | N (PPT/PPS) |
| --- | --- | --- | --- | --- |
| Month 1 | -2.15 ± 0.75 (-2.55, -1.75) | -2.41 ± 0.60 (-2.73, -2.09) | 0.26 (-0.23, 0.76) | 16/16 |
| Month 3 | -0.51 ± 0.43 (-0.74, -0.28) | -0.60 ± 0.43 (-0.83, -0.37) | 0.09 (-0.22, 0.40) | 16/16 |

Notes: Means with t-based 95% CIs are descriptive; Δ denotes within-group change from baseline; ΔΔ denotes between-group difference in change with Welch 95% CI.
