# Supplementary material for: Comparative analysis of onabotulinum toxin type-A injection techniques in older adults with blepharospasm: a retrospective cohort study
Source: Front Neurol. 2025 Oct 17;16:1601911. doi: 10.3389/fneur.2025.1601911 (PMC12576801; doi:10.3389/fneur.2025.1601911)
Supplement: Supplementary file 2 [file Table_2.docx]

**Table S2. mJS-S — Estimated means and changes by Group × Time**

| Group | Baseline mean ± SD (95% CI) | Month 1 mean ± SD (95% CI) | Month 3 mean ± SD (95% CI) | N |
| --- | --- | --- | --- | --- |
| PPT | 2.56 ± 0.96 (2.05, 3.08) | 0.56 ± 0.63 (0.23, 0.90) | 2.00 ± 0.73 (1.61, 2.39) | 16 |
| PPS | 2.50 ± 0.63 (2.16, 2.84) | 0.12 ± 0.34 (-0.06, 0.31) | 1.81 ± 0.66 (1.46, 2.16) | 16 |

| Timepoint | Δ (PPT) mean ± SD (95% CI) | Δ (PPS) mean ± SD (95% CI) | ΔΔ (PPT − PPS) (95% CI) | N (PPT/PPS) |
| --- | --- | --- | --- | --- |
| Month 1 | -2.00 ± 0.73 (-2.39, -1.61) | -2.38 ± 0.62 (-2.70, -2.05) | 0.38 (-0.11, 0.86) | 16/16 |
| Month 3 | -0.56 ± 0.51 (-0.84, -0.29) | -0.69 ± 0.48 (-0.94, -0.43) | 0.12 (-0.23, 0.48) | 16/16 |

Notes: Means with t-based 95% CIs are descriptive; Δ denotes within-group change from baseline; ΔΔ denotes between-group difference in change with Welch 95% CI.
