# Supplementary material for: Comparative analysis of onabotulinum toxin type-A injection techniques in older adults with blepharospasm: a retrospective cohort study
Source: Front Neurol. 2025 Oct 17;16:1601911. doi: 10.3389/fneur.2025.1601911 (PMC12576801; doi:10.3389/fneur.2025.1601911)
Supplement: Supplementary file 3 [file Table_3.docx]

**Table S3. mJS-F — Estimated means and changes by Group × Time**

| Group | Baseline mean ± SD (95% CI) | Month 1 mean ± SD (95% CI) | Month 3 mean ± SD (95% CI) | N |
| --- | --- | --- | --- | --- |
| PPT | 2.75 ± 0.68 (2.39, 3.11) | 0.31 ± 0.48 (0.06, 0.57) | 1.94 ± 0.68 (1.58, 2.30) | 16 |
| PPS | 2.81 ± 0.54 (2.52, 3.10) | 0.44 ± 0.51 (0.16, 0.71) | 2.19 ± 0.54 (1.90, 2.48) | 16 |

| Timepoint | Δ (PPT) mean ± SD (95% CI) | Δ (PPS) mean ± SD (95% CI) | ΔΔ (PPT − PPS) (95% CI) | N (PPT/PPS) |
| --- | --- | --- | --- | --- |
| Month 1 | -2.44 ± 0.63 (-2.77, -2.10) | -2.38 ± 0.50 (-2.64, -2.11) | -0.06 (-0.47, 0.35) | 16/16 |
| Month 3 | -0.81 ± 0.40 (-1.03, -0.60) | -0.62 ± 0.50 (-0.89, -0.36) | -0.19 (-0.52, 0.14) | 16/16 |

Notes: Means with t-based 95% CIs are descriptive; Δ denotes within-group change from baseline; ΔΔ denotes between-group difference in change with Welch 95% CI.
