# Supplementary material for: Comparative analysis of onabotulinum toxin type-A injection techniques in older adults with blepharospasm: a retrospective cohort study
Source: Front Neurol. 2025 Oct 17;16:1601911. doi: 10.3389/fneur.2025.1601911 (PMC12576801; doi:10.3389/fneur.2025.1601911)
Supplement: Supplementary file 4 [file Table_4.docx]

**Table S4. Schirmer I (mm) — Estimated means and changes by Group × Time**

| Group | Baseline mean ± SD (95% CI) | Month 1 mean ± SD (95% CI) | Month 3 mean ± SD (95% CI) | N |
| --- | --- | --- | --- | --- |
| PPT | 8.59 ± 4.41 (6.24, 10.95) | 13.00 ± 4.03 (10.85, 15.15) | 11.09 ± 3.87 (9.03, 13.15) | 16 |
| PPS | 7.50 ± 3.04 (5.88, 9.12) | 11.81 ± 2.97 (10.23, 13.39) | 9.56 ± 2.91 (8.01, 11.11) | 16 |

| Timepoint | Δ (PPT) mean ± SD (95% CI) | Δ (PPS) mean ± SD (95% CI) | ΔΔ (PPT − PPS) (95% CI) | N (PPT/PPS) |
| --- | --- | --- | --- | --- |
| Month 1 | 4.41 ± 2.03 (3.32, 5.49) | 4.31 ± 1.97 (3.26, 5.36) | 0.09 (-1.35, 1.54) | 16/16 |
| Month 3 | 2.50 ± 1.88 (1.50, 3.50) | 2.06 ± 1.55 (1.24, 2.89) | 0.44 (-0.81, 1.68) | 16/16 |

Notes: Means with t-based 95% CIs are descriptive; Δ denotes within-group change from baseline; ΔΔ denotes between-group difference in change with Welch 95% CI.
