# Supplementary material for: Comparative analysis of onabotulinum toxin type-A injection techniques in older adults with blepharospasm: a retrospective cohort study
Source: Front Neurol. 2025 Oct 17;16:1601911. doi: 10.3389/fneur.2025.1601911 (PMC12576801; doi:10.3389/fneur.2025.1601911)
Supplement: Supplementary file 6 [file Table_6.docx]

**Table S6. Category distribution of Modified Jankovic Scale–Frequency (mJS-F) by Group and Time**

| Time | Group | N | 0 n (%) | 1 n (%) | 2 n (%) | 3 n (%) | 4 n (%) |
| --- | --- | --- | --- | --- | --- | --- | --- |
| Baseline | PPT | 16 | 0 (0.0%) | 0 (0.0%) | 6 (37.5%) | 8 (50.0%) | 2 (12.5%) |
| Baseline | PPS | 16 | 0 (0.0%) | 0 (0.0%) | 4 (25.0%) | 11 (68.8%) | 1 (6.2%) |
| Month 1 | PPT | 16 | 11 (68.8%) | 5 (31.2%) | 0 (0.0%) | 0 (0.0%) | 0 (0.0%) |
| Month 1 | PPS | 16 | 9 (56.2%) | 7 (43.8%) | 0 (0.0%) | 0 (0.0%) | 0 (0.0%) |
| Month 3 | PPT | 16 | 0 (0.0%) | 4 (25.0%) | 9 (56.2%) | 3 (18.8%) | 0 (0.0%) |
| Month 3 | PPS | 16 | 0 (0.0%) | 1 (6.2%) | 11 (68.8%) | 4 (25.0%) | 0 (0.0%) |
